# The Home Treatment of Hay Fever1Read before the Central New York Medical Association, Buffalo, N. Y., October 16, 1894.

**Published:** 1895-01

**Authors:** A. L. Hall

**Affiliations:** Fair Haven, N. Y.


### THE HOME TREATMENT OF HAY FEVER.[1]

By A. L. HALL, M. D., Fair Haven, N. Y.

IN THE present state of our knowledge it may be safely asserted that hay fever is a respiratory neurosis whose essential dependence is a neurasthenic constitution. The other etiological factors—an irritable state of the respiratory mucosa and the various kinds of stimulus—occupy a secondary place and without the presence of the neurotic element are incapable of producing the aggregation of symptoms known as hay fever.

Accepting the theory of the preëminence of the nervous element as a causative factor of the affection obviously, then, the first indication for intelligent treatment must include such hygienic and therapeutic measures as are calculated to improve and preserve the stability of the nervous system. Among the chief hygienic conditions to be observed are the following: an orderly manner of living, including appropriate diet and well-ventilated apartments, the avoidance of physical and mental overstrain—depressing influences—and exposure to dust, pollen and other external excitants. Among the therapeutical agents which promote reconstructive metamorphosis and conserve nervous energy, arsenic, nux vomica and cinchona, taken in the order named, are of the highest value, and will be found the most serviceable remedies for the removal of the neurotic condition which underlies hay fever. The potentiality of these drugs are materially enhanced by combining them. The truth of this assertion may be well determined

1. Read before the Central New York Medical Association, Buffalo, N. Y., October 16, 1894.

by carefully noting the effects derived from their use according to the following formula:

℞ Lig. pot. arsenitis.
Ext. nucis vomicæ fl.
Ext. cinchona fl. (detannated) .............. aa vi. drs.
Alcoholis ...................................... iii ozs.
Syrp. aurantii........................ q. s. ad. xvi. ozs.

M. Sig.—One to two teaspoonfuls, taken three times daily, with or after meals.

To be eminently successful, the use of this mixture should be commenced at least six weeks before the time for the annual recurrence of the disease and should be faithfully maintained for the ensuing three months, or longer if demanded. This treatment, aided by proper hygienic conditions, will gradually lessen the severity of the affection, and in a comparatively short time bring the patient into a comfortable state of existence. Especially noteworthy will be the relief obtained from the naso-pharyngeal and bronchial dyspnea which is such an annoying symptom of the disease. Furthermore, there are good reasons for believing that from three to five years' consecutive treatment, covering the three months' period named, will, in many cases, secure permanent immunity from subsequent attacks of the affection. In support of these claims, the history and results of treatment in two cases are offered.

Case I.—Female, aged 42 years; neurotic. Father and paternal grandfather asthmatic. Thirteen years ago experienced first attack of hay fever. Came on during latter part of August and lasted eight weeks. Commenced with well-marked coryza accompanied with severe nasal dyspnea and considerable bronchial catarrh. For the succeeding five years there was no recurrence of the attacks. Then, for four successive seasons, the attacks were renewed and assumed unusual violence, all of the symptoms being intensified, severe asthmatic paroxysms resulting, which, at one time, were present for a considerable portion of sixteen successive days, and for the relief of which nearly all the various asthmatic remedies were tried, with anything but encouraging results. Three years ago the patient determined to find immunity from the disease by spending the period of the annual attack in some exempt locality. To carry out her determination was found to be impracticable, owing to family matters, and it was then decided that she should be put upon a thorough course of treatment, involving the use of the nerve tonics, aided by proper hygienic conditions. This treatment was instituted in the early part of the month of July, 1891, and was continued for three months, the remedies indicated in the formula given being the principal therapeusis employed. A prompt response to these measures followed, the strength and bodily weight were increased and a general feeling of well-being ensued. In the last week of August there occurred a mild accession of hay fever symptoms which continued for about four weeks. Throughout the entire attack there was no asthma, although some bronchial irritation was present. Irritation of the naso-pharyngeal mucosa was very much less than it had been in previous attacks, and the nasal dyspnea was milder and much shorter in duration. In fact, the sense of relief was so great that the patient gave up the desire to seek a more congenial climate, and for the past three years has followed the treatment with increasing satisfaction, her hay fever being reduced to the form of a mild coryza without asthma, and with only an infrequent sense of impending nasal dyspnea. Some other measures were tried in this and other cases for the relief of nasal and pulmonary asthma with considerable success, such as blistering between the scapulæ, the inhalation of warm creasote vapor, the use of smelling salts composed of equal parts of menthol and carbonate of ammonia, and vigorous rubbing of the integument of the upper part of the face and the scalp with a flesh brush, not omitting to give the ears a proper amount of brisk rubbing, as recommended by the American Hay Fever Association. Oftentimes, by its derivative action, the last-named expedient temporarily relieves the nasal dyspnea by overcoming the vaso-motor turgescence of the upper air-passages upon which the stenosis depends.

CASE II.—Female, aged 47 years; wife of well-to-do farmer; nervous temperament. Three years ago last August first attack of hay fever occurred. Had characteristic coryza, with paroxysms of nasal and pulmonary asthma. The attack lasted about two months, and recurred at the usual time for the next two years when the asthma habit was acquired, the paroxysms occurring at all seasons of the year, from either exposure to cold or mental excitement. On the twenty-second day of January, 1894, she was placed upon the tonic treatment, supplemented for a few days with sedatives for arresting the asthmatic paroxysms then in active progress. The tonic has been taken continuously, with only slight interruption, from that date until the present time, with the effect of completely annulling the asthmatic paroxysms as well as affording almost entire relief from the other symptoms. A transitory coryza appeared for a few days during the early part of September, which has been the only noticeable symptom of the disease. There has also been very marked improvement in the general health and a very considerable increase of the bodily weight, and she is now able to perform her ordinary household duties with comparative comfort. Since last January she has had no other treatment except as has been mentioned herein, but for a period of two years prior thereto she tried many remedies without obtaining special relief.

In conclusion it may be truthfully inferred that the results obtained in these two cases are only fairly illustrative of the good effects obtainable from the persistent use of the remedies and other measures indicated herein; while, furthermore, this treatment is apparently capable of securing greater relief from the distressing symptoms of hay fever than are derived from the operative procedures for the destruction of the hypersensitive areas of the nasopharyngeal mucous membrane, so much in vogue, without loss of time, absence from home or business or other disagreeable consequence. For these reasons the plan has been very properly termed the home treatment of hay fever, and will, if faithfully executed, sustain the claims made for it.

DISCUSSION.

Dr. J. O. Roe: In the production of hay fever we recognize the fact that we must have three factors. We must first have the local irritant; then we must have the spot that is locally irritated, and we usually have a susceptible nervous system that communicates this local irritation to the general system. Now, the simplest method of treatment of hay fever would, of course, be to remove the local irritant. The next step would be to remove the point of local irritation and at the same time to build up the system and render the nervous system less susceptible to nervous irritation. Now, by the plan of treatment which the doctor has advocated he directs it entirely to the treatment of one side of the question, simply to building up the nervous system so that it will not communicate the affection from the nasal to the other portions of the body. I do not understand why, in order to treat this subject scientifically, the treatment of the disease may not be considered a local treatment just as much as to treat the general system which is irritated with this disease. That is the plan of treatment which is most susceptible of good results, in my opinion.

Dr. Hall: I fully recognized at the outset of my paper the three essential conditions upon which hay fever depends, the same as outlined by the last speaker. We agree in the main, but differ regarding the relative importance of these conditions, especially in their bearing upon the treatment. I maintain that the neurotic condition is the element of primary importance in all cases of the disease, without which it cannot originate or exist, and is, therefore, the chief object for removal. With some adroitness, the doctor arrives at the conclusion that the primary condition is always the point of local irritation and with its removal the active cause subsides and the disease must necessarily come to an end—aided, it may be, by the action of general remedies. I have reason for inferring that in removing the "point of local irritation," the nasal saw or its equivalent is the measure employed while a minimum of general remedies are given. As the result of my observation, I believe that less cases are cured by the removal of the local irritation than are cured by the general treatment that removes the neurotic element. Moreover, many patients will not submit to the operative treatment, while there are others who are unable to leave home or bear the expense of special treatment. To this class of patients the treatment I have presented offers as good results as can be obtained, even at the hands of the "specialist," and as such should receive the support and encouragement of the general practitioner.

